# Unveiling metabolic integration in psyllids and their nutritional endosymbionts through comparative transcriptomics analysis

**DOI:** 10.1016/j.isci.2023.107930

**Published:** 2023-09-15

**Authors:** Younghwan Kwak, Allison K. Hansen

**Affiliations:** 1 Department of Life and Environmental Sciences, University of California, Merced, 5200 Lake Road, Merced, CA 95343, USA; 2 Department of Entomology, University of California, Riverside, 900 University Avenue, Riverside, CA 92521, USA

**Keywords:** Microbiology, Phylogenetics, Transcriptomics

## Abstract

Psyllids, a group of insects that feed on plant sap, have a symbiotic relationship with an endosymbiont called *Carsonella*. *Carsonella* synthesizes essential amino acids and vitamins for its psyllid host, but lacks certain genes required for this process, suggesting a compensatory role of psyllid host genes. To investigate this, gene expression was compared between two psyllid species, *Bactericera cockerelli* and *Diaphorina citri*, in specialized cells where *Carsonella* resides (bacteriomes). Collaborative psyllid genes, including horizontally transferred genes, showed patterns of conserved gene expression; however, species-specific patterns were also observed, suggesting differences in the nutritional metabolism between psyllid species. Also, the recycling of nitrogen in bacteriomes may primarily rely on glutamate dehydrogenase (*GDH*). Additionally, lineage-specific gene clusters were differentially expressed in *B. cockerelli* and *D. citri* bacteriomes and are highlighted here. These findings shed light on potential host adaptations for the regulation of this symbiosis due to host, microbiome, and environmental differences.

## Highlights

• Psyllids exhibit divergent and conserved gene expression patterns in bacteriomes
• All nutritional HTG copies are upregulated for both psyllid species in bacteriomes
• The recycling of ammonia in bacteriomes is via the GS and GDH pathway
• Sulfur, cysteine, and methionine metabolisms enriched only in *D. citri* bacteriomes

## Graphical abstract

**Figure:**
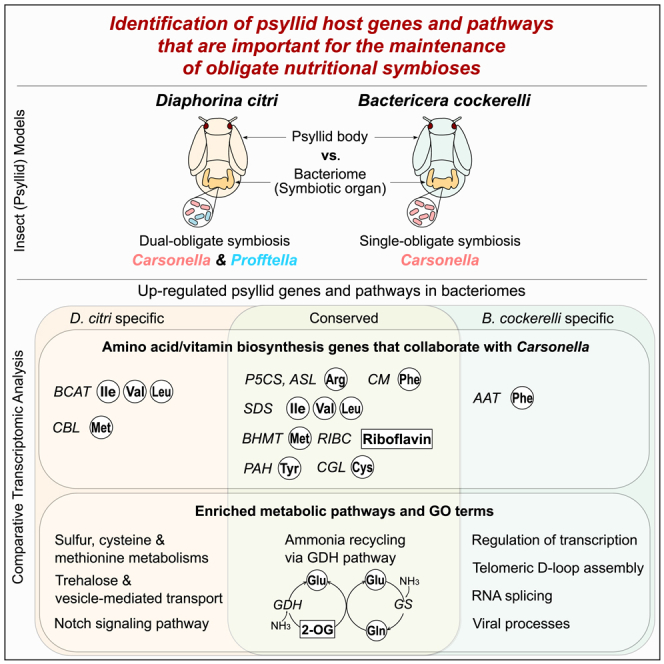

## Introduction

A multitude of insects have forged mutualistic relationships with microbial symbionts, thereby enhancing their metabolic capabilities and tolerance to diverse environmental conditions. These insects rely on symbionts to supplement their nutrition and aid in the digestion of plant materials, enabling them to thrive in their respective habitats.^1^ These associations require specific genetic traits in insects, encompassing gene expression patterns in cells hosting symbionts,^2^ gene duplication,^3^^,^^4^^,^^5^ and horizontal gene transfer.^6^^,^^7^^,^^8^ These insect traits play a pivotal role in the development and maintenance of obligatory symbiotic mutualisms.

Psyllids, sap-feeding insects in the order Hemiptera, serve as an exemplary illustration of such mutualisms. They uphold an obligate symbiotic relationship with *Candidatus* Carsonella ruddii (referred to as *Carsonella* hereafter), which synthesizes vital amino acids that are typically scarce in plant sap.^9^^,^^10^^,^^11^^,^^12^^,^^13^^,^^14^ *Carsonella* resides within specialized cells called bacteriocytes, which are found in the abdominal organ known as the bacteriome.^15^^,^^16^ This long-term, host-restricted relationship leads to continuous gene loss in *Carsonella*’s genome because of genetic drift.^11^^,^^14^^,^^17^^,^^18^ Research conducted on *Carsonella* and the hackberry petiole gall psyllid, *Pachypsylla venusta*, has unveiled that a distinct subset of psyllid genes is up-regulated in bacteriomes, as compared to other body tissues.^7^ This finding suggests a potential compensation mechanism for *Carsonella*’s gene loss and the establishment of integrated host-microbe metabolic pathways, similar to those observed in other hemipteran species.^2^^,^^6^^,^^19^ Nonetheless, the extent of functional and expression divergence among these collaborative genes, including horizontally transferred genes (HTGs), remains uncertain across divergent psyllid species.

Recently, chromosomal-level genome assemblies have been successfully obtained for three divergent psyllid species: *P. venusta* (Carsidaridae),^20^ the Asian citrus psyllid *Diaphorina citri* (Psyllidae),^21^ and the potato/tomato psyllid *Bactericera cockerelli* (Triozidae).^22^ *Bactericera cockerelli* and *D. citri* diverged from each other approximately 86 million years ago, while their most recent common ancestor separated from *P. venusta* around 130 million years ago.^22^ Our previous comparative genomic analysis has revealed that these three psyllid species share notable homologs which could potentially compensate for *Carsonella*’s incomplete amino acid biosynthesis pathways, specifically in six essential amino acid pathways.^22^ Remarkably, some of these homologs have been acquired through horizontal gene transfer events from bacteria to an early ancestor of psyllids before the major divergence of the Psylloidea superfamily.^7^^,^^22^ While these HTGs are largely conserved among the three psyllid species, lineage-specific gene duplication events have also been observed.^22^ Nevertheless, It remains uncertain whether divergent psyllid species exhibit similar expression patterns in bacteriomes for the homologs that are predicted to collaborate with *Carsonella* in essential amino acid biosynthesis, as the expression patterns of psyllid bacteriomes have only been examined for one psyllid species to date (*P. venusta)*.^7^

In this study, we present bacteriome and body transcriptomic data for two additional psyllid species *D. citri* and *B. cockerelli*. Furthermore, we conduct inter-species comparative transcriptomic analyses, aiming to enhance our understanding of the evolutionary aspects of psyllid host regulation. In comparison to *B. cockerelli, D. citri* hosts an additional obligate bacterial endosymbiont known as *Candidatus* Profftella armatura (hereafter referred to as *Profftella*), which resides in the syncytial region of the bacteriome.^23^ *Profftella* is presumed to primarily serve as a defensive symbiont for the psyllid by producing a polyketide toxin called diaphorin, but its genome also contains genes related to the biosynthesis of cysteine, hemolysin, riboflavin, biotin, and carotenoids, which may contribute to *D. citri*’s nutritional symbiosis.^23^^,^^24^ Both psyllid species also harbor *Wolbachia*,^25^^,^^26^ one of the most prevalent facultative endosymbionts of arthropods,^27^ including psyllids.^26^^,^^28^^,^^29^^,^^30^^,^^31^ The impact of *Wolbachia* on the nutritional metabolism of the psyllid host-symbiont relationship is also currently unknown. Therefore, It is of significant interest to investigate the following questions: 1) To what extent are gene expression patterns conserved between *D. citri* and *B. cockerelli* bacteriomes for psyllid homologs that collaborate with *Carsonella*?, 2) Are there species-specific gene expression patterns within *D. citri* and *B. cockerelli* bacteriomes, suggesting divergence in their nutritional symbioses?, and 3) Do psyllid bacteriomes exhibit distinct gene expression patterns for homologs with multiple gene copies, such as the horizontally transferred genes (HTGs) predicted to collaborate with *Carsonella*? By examining these questions, we can gain deeper insights into the evolution of psyllid host regulation and the intricate dynamics of their nutritional symbioses.

## Results

### Global differential gene expression in bacteriomes of *D. citri* and *B. cockerelli*

To investigate conserved and lineage-specific gene expression patterns related to the nutritional symbiosis in two psyllid species, *D. citri* and *B. cockerelli,* we pooled 60 bacteriomes and the rest of the body tissues with three biological replicates each for each species (N = 12 samples; Figure S1; see STAR methods for more detail). For *D. citri*, we obtained an average of 24,621,735 and 24,442,844 high-quality trimmed reads from three biological replicates of bacteriomes and body tissues, respectively, with an average of 75% and 89% of these reads mapping to the *D. citri* genome, respectively (Table S1). For *B. cockerelli*, we obtained an average of 26,365,738 and 27,932,791 trimmed reads from three biological replicates of bacteriomes and body tissues, respectively, with an average of 80% and 76% of these reads mapping to the *B. cockerelli* genome, respectively (Table S1).

In *D. citri*, for a total of 23,078 genes, 3,673 (16%) and 4,112 (18%) genes were significantly up- and down-regulated, respectively, in bacteriomes compared to the body tissues (Tables S2A and S3A). In *B. cockerelli*, for a total of 19,032 genes, 4,589 (24%) and 3,982 (21%) genes were significantly up- and down-regulated, respectively, in bacteriomes compared to the body tissues (Tables S2B and S3B).

### Inter-species comparison of one-to-one orthologs

**Figure 1:**
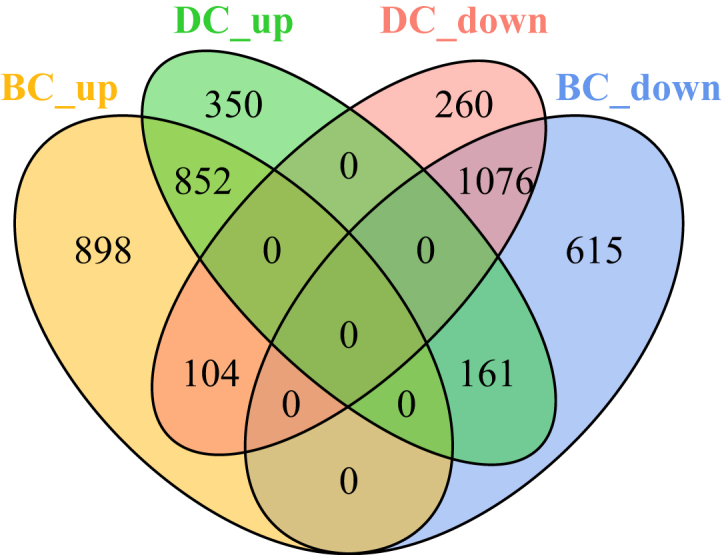
A Venn diagram showing the number of significantly differentially expressed one-to-one orthologs in *D. citri* and *B. cockerelli* for bacteriomes compared to body tissues (FDR adjusted p value ≤0.05 and fold change (FC) ≥ 1.5) Four sets of significantly differentially expressed one-to-one orthologs are represented (from left to right; up-regulated one-to-one orthologs in *B. cockerelli* (BC_up; outlined in yellow), up-regulated one-to-one orthologs in *D. citri* (DC_up; outlined in green), down-regulated one-to-one orthologs in *D. citri* (DC_down; outlined in pink), and down-regulated one-to-one orthologs in *B. cockerelli* (BC_down; outlined in blue).

When comparing one-to-one orthologs (N = 5,555) between both psyllid species (Table S4), we identified an overlap of 852 orthologs (15%) that were significantly up-regulated in bacteriomes compared to body for both psyllid species, while 511 and 1,002 orthologs were significantly up-regulated in only *D. citri* or *B. cockerelli*, respectively (Figure 1; Table S4). The most represented GO-terms for shared up-regulated one-to-one orthologs include metal ion binding, ubiquitin-dependent protein catabolic process, protein transport, and transmembrane transport. The most represented GO-terms for one-to-one orthologs with species-specific up-regulation patterns include trehalose transport and vesicle-mediated transport for *D. citri*, and regulation of transcription, RNA splicing and viral processes for *B. cockerelli* (Table S4). For significantly down-regulated genes in bacteriomes compared to body for both psyllid species there was an overlap of 1,076 orthologs (19%), whereas 364 and 776 orthologs were significantly down-regulated in only *D. citri* or *B. cockerelli*, respectively (Figure 1; Table S4). The most represented GO-terms for shared down-regulated one-to-one orthologs include signal transduction, visual perception, integral component of membrane and multicellular organism development. The most represented GO-terms for one-to-one orthologs with species-specific down-regulation include zinc ion binding, response to oxidative stress and Wnt signaling pathway for *D. citri* and translation, ventral cord development and cell adhesion for *B. cockerelli* (Table S4).

To further explore the similarities and differences in gene expression between *D. citri* and *B. cockerelli,* we conducted Principal Component Analyses (PCA) on the relative expression of one-to-one orthologs similar to Argandona et al. (2023),^32^ Georgiadou et al. (2022)^33^ and Korb et al. (2021).^34^ Approximately 39% of one-to-one orthologs were significantly differentially expressed in bacteriomes compared to the body tissues for both psyllid species and these genes were examined further using PCA analysis where axis 1 and 2 explained ∼51% and 48% of the variance in the data, respectively. Out of the top 100 orthologs that displayed the highest positive and negative correlations with principal components 1 and 2, 67 of these genes were non-redundant and examined further. These orthologs consist of 43 up-regulated and 24 down-regulated genes in *B. cockerelli* and 41 up-regulated and 26 down-regulated genes in *D. citri* (Figure S2; Table S5).

Out of the 67 orthologs, those that are involved in a horizontal gene transfer event and symbiosis include the rRNA methylation gene (*RSMJ*) which was up-regulated in both species in bacteriomes compared to the body tissues (Tables 2 and S5). Moreover, up-regulated orthologs appear to have two distinct expression profiles with a subset of orthologs having greater (or lower) FC magnitudes in either *D. citri* or *B. cockerelli* (Figure S2). For instance, orthologs that were associated with the sulfur compound metabolism and ubiquitin binding showed higher up-regulation profiles in *D. citri*, while orthologs that were associated with rRNA-methyltransferase activity, trigeminal ganglion development exhibited higher up-regulation profiles in *B. cockerelli* (Figure S2; Table S5). Down-regulated genes in both species include those associated with thermotaxis, bursicon, and the regulation of sleep (protein quiver/sleepless) (Figure S2; Table S5).

### Species-specific gene expression patterns are prevalent for collaborative genes in the essential amino acid metabolism

**Figure 2:**
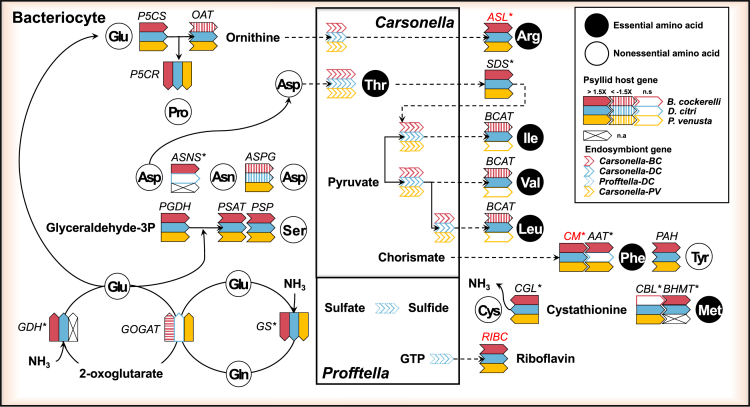
Differential gene expression of psyllid genes involved in the integrative metabolism with *Carsonella* and *Profftella* Gene expression data are from NCBI SRA: SRA099681 for *Pachypsylla venusta,* and from this study for *B. cockerelli* and *D. citri*. Asterisks indicate more than one homolog is present in *B. cockerelli* or *D. citri*. HTGs are shown in red. (>1.5X, significantly up-regulated; <-1.5X, significantly down-regulated; n.s., not significant; n.a. data not available from *P. venusta*).

To gain insights into the mechanisms by which divergent psyllid hosts maintain their mutualistic relationship with *Carsonella* (as well as *Profftella*), we explored the differential expression patterns of psyllid host genes in the bacteriomes compared to other body tissues. This investigation aimed to shed light on the hypothesized role of certain psyllid genes in collaborating with *Carsonella* for the biosynthesis of essential amino acids, including arginine, phenylalanine, isoleucine, leucine, valine, and methionine (Figure 2).^7^^,^^22^ Three psyllid homologs hypothesized to be involved in the arginine, isoleucine, and methionine pathway, such as 1-pyrroline-5-carboxylate synthase (*P5CS*, EC 2.7.2.11/1.2.1.41), L-serine/L-threonine ammonia-lyase (*SDS*, EC 4.3.1.17/4.3.1.19), and homocysteine S-methyltransferase (*BHMT*, EC 2.1.1.10), respectively, were all significantly up-regulated in bacteriomes compared to the body tissues (Table 1; Figure 2), suggesting that both psyllid hosts may complement *Carsonella*’s biosynthesis pathways for arginine, isoleucine, and methionine biosynthesis.

**Table 1:** Expression of symbiosis-related homologs in bacteriomes compared to the body tissues for *D. citri* and *B. cockerelli*

Name	EC Number	Enzyme	*B. cockerelli* gene ID	LogFC	FDR	*D. citri* gene ID	LogFC	FDR
**Collaborative essential amino acid genes**								

*P5CS*	2.7.2.11/1.2.1.41	delta-1-pyrroline-5-carboxylate synthetase	**ANN07147**	3.74	1.36E-16	**M8J77_013564**	5.49	1.33E-51
*OAT*	2.6.1.13	ornithine—oxo-acid transaminase	**ANN07991**	−1.19	5.46E-05	**M8J77_012790**	5.63	1.28E-39
*BCAT*	2.6.1.42	branched-chain amino acid aminotransferase	**ANN10973**	−0.87	0.02	**M8J77_022158**	0.93	0.00
*SDS*	4.3.1.17/4.3.1.19	L-serine/L-threonine ammonia-lyase	**ANN22278**	6.23	6.25E-11	N/A	N/A	N/A
			N/A	N/A	N/A	**M8J77_020702**	1.04	0.01
						**M8J77_019021**	9.68	2.43E-128
*AAT*	2.6.1.1		**ANN17803**	1.51	0.00	M8J77_007026	0.17	0.43
						M8J77_014008	−0.22	0.76
						M8J77_009315	0.55	0.08
*CBL*	4.4.1.13	cysteine-S-conjugate beta-lyase	ANN16046	0.16	0.47	**M8J77_020257**	1.19	0.01
						M8J77_023431	0.14	0.57
			ANN16088	0.51	0.00	**M8J77_004574**	0.72	0.03
						M8J77_012408	0.50	0.45
*BHMT*	2.1.1.10	homocysteine S-methyltransferase	**ANN06058**	−1.36	9.56E-09	M8J77_021131	0.49	0.04
			**ANN06059**	3.55	1.13E-59	**M8J77_014773**	5.70	1.11E-82

**Non-essential amino acid psyllid genes**								

*GOGAT*	1.4.1.13	Glutamate synthase (NADH)	**ANN19375**	−0.75	0.02	M8J77_004711	0.57	0.02
*GDH*	1.4.1.3	glutamate dehydrogenase	**ANN04489**	−1.43	5.39E-10	**M8J77_003227**	−0.94	3.63E-06
			**ANN10746**	7.77	0.00	**M8J77_018416**	5.48	0.01
*GS*	6.3.1.2	glutamine synthetase	**ANN10146**	1.69	8.73E-05	**M8J77_024675**	3.08	4.63E-39
			**ANN10148**	1.85	2.92E-06			
*PAH*	1.14.16.1	phenylalanine-4-hydroxylase	**ANN09753**	1.92	2.03E-06	**M8J77_010704**	5.54	1.51E-55
*ASNS*	6.3.5.4	Asparagine synthase	**ANN02335**	1.75	5.62E-12	N/A	N/A	N/A
			**ANN09881**	1.68	3.20E-26	M8J77_011885	−0.17	0.47
			**ANN21723**	1.65	3.94E-07	M8J77_022917	−0.13	0.66
*ASPG*	3.5.1.1	L-asparaginase	**ANN07324**	−2.71	1.29E-12	**M8J77_015442**	−1.54	0.00
*P5CR*	1.5.1.2	pyrroline-5-carboxylate reductase	**ANN22280**	2.96	4.31E-17	N/A	N/A	N/A
			N/A	N/A	N/A	M8J76_017158	−0.50	0.28
						**M8J77_004141**	5.88	6.60E-76
*CGL*	4.4.1.1	Cystathionine gamma lyase	**ANN04485**	4.96	2.95E-38	**M8J77_022568**	6.66	1.26E-85
			**ANN04499**	5.27	1.64E-34	N/A	N/A	N/A
*PGDH*	1.1.1.95	Phosphoglycerate dehydrogenase	**ANN19564**	2.76	1.00E-37	**M8J77_016165**	4.66	8.83E-39
*PSAT*	2.6.1.52	Phosphoserine aminotransferase	**ANN12533**	3.35	7.73E-82	**M8J77_018069**	2.95	2.71E-29
*PSP*	3.1.3.3	Phosphoserine phosphatase	**ANN22282**	6.18	2.97E-29	N/A	N/A	N/A
			N/A	N/A	N/A	**M8J77_025516**	6.09	4.14E-77

Homologs in the same row are one-to-one or one to many orthologs. “N/A” indicates no ortholog was identified. Bolded gene IDs indicate that the psyllid gene is significantly differentially expressed in bacteriomes compared to the body tissues.

In contrast, the ortholog ornithine aminotransferase (*OAT*, EC 2.6.1.13), which provides an alternative pathway to synthesize ornithine via *P5CS* in the arginine pathway, was significantly up-regulated in *D. citri* and significantly down-regulated in *B. cockerelli* (Table 1; Figure 2). Species-specific expression patterns were also observed for other collaborative psyllid homologs in the phenylalanine, methionine, and branched-chain amino acid biosynthesis pathways (e.g., leucine, valine, and isoleucine), suggesting distinct strategies in collaborating with *Carsonella* to synthesize essential amino acids. For instance, aspartate aminotransferase (*AAT*, EC 2.6.1.1) was significantly up-regulated in bacteriomes compared to body tissues only in *B. cockerelli* for phenylalanine synthesis. Cysteine-S-conjugate beta-lyase (*CBL*, EC 4.4.1.13) was only significantly up-regulated in bacteriomes compared to body tissues in *D. citri* for the methionine pathway, and branched-chain aminotransferase (*BCAT*, EC 2.6.1.42) was significantly up- and down-regulated in bacteriomes compared to body tissues in *D. citri* and *B. cockerelli*, respectively, for the biosynthesis of leucine, valine, and isoleucine (Table 1; Figure 2).

### Expression patterns of psyllid genes in the non-essential amino acid metabolism

The two enzymes, glutamine synthetase (*GS*, EC 6.3.1.2) and glutamine oxoglutarate aminotransferase (*GOGAT*, EC 1.4.1.13), are hypothesized to work together in recycling waste ammonia (NH_3_) in aphid bacteriocytes into glutamine and glutamate, which are used as amino donors for amino acid biosynthesis.^2^ Recycling of ammonia is crucial for sap-feeding insects due to the limited availability of nitrogen in their diets.^35^ Here in both *B. cockerelli* and *D. citri*, we found all *GS* orthologs were up-regulated in bacteriomes compared to the body tissues, while species-specific expression patterns for the *GOGAT* ortholog was observed (Table 1; Figure 2). The *GOGAT* ortholog in *B. cockerelli* was significantly down-regulated in bacteriomes compared to the body tissues whereas this ortholog was not significantly differentially expressed in *D. citri* (Table 1; Figure 2). Interestingly, orthologs for glutamate dehydrogenase (*GDH*, EC 1.4.1.3), an alternative to the GS/GOGAT pathway for recycling ammonia into glutamate, were significantly up-regulated in bacteriomes compared to body tissues for both psyllid species (Table 1; Figure 2).

The activity of asparaginase (*ASPG*, EC 3.5.1.1), that converts asparagine to aspartate, has been hypothesized to be a primary source of ammonia for the GS/GOGAT cycle.^6^^,^^7^^,^^36^ Here, we found that the *ASPG* ortholog in both psyllid species were significantly down-regulated in bacteriomes compared to the body tissues (Table 1; Figure 2). However, there were species-specific expression patterns for the biosynthesis of asparagine via asparagine synthase (*ASNS*, EC 6.3.5.4), where all three homologs in *B. cockerelli* were significantly up-regulated in bacteriomes compared to the body tissues, however, the two *ASNS* genes identified in *D. citri* were not significantly differentially expressed (Table 1; Figure 2).

In regard to other non-essential amino acid biosynthesis pathways, such as tyrosine, cysteine, proline, and serine biosynthesis, we observed that the majority of homologs for both psyllid species were significantly up-regulated in bacteriomes compared to the body tissues. For instance, phenylalanine-4-hydroxylase (*PAH*, EC 1.14.16.1) in the tyrosine pathway, cystathionine gamma lyase (*CGL*, EC 4.4.1.1) in the cysteine pathway, which contributes to ammonia production, pyrroline-5-carboxylate reductase (*P5CR*, EC 1.5.1.2) in the proline pathway, and three genes in the serine pathway such as phosphoglycerate dehydrogenase (*PGDH*, EC 1.1.1.95), phosphoserine aminotransferase (*PSAT*, EC 2.6.1.52), and phosphoserine phosphatase (*PSP*, EC 3.1.3.3), were significantly up-regulated in bacteriomes compared to body tissues for both psyllid species (Table 1; Figure 2). These findings indicate a significant involvement of the psyllid host in the bacteriome of both species, for the biosynthesis of tyrosine, cysteine, proline, and serine.

### Horizontal gene transfer events in psyllid genomes may support gene losses in *Carsonella*

**Figure 3:**
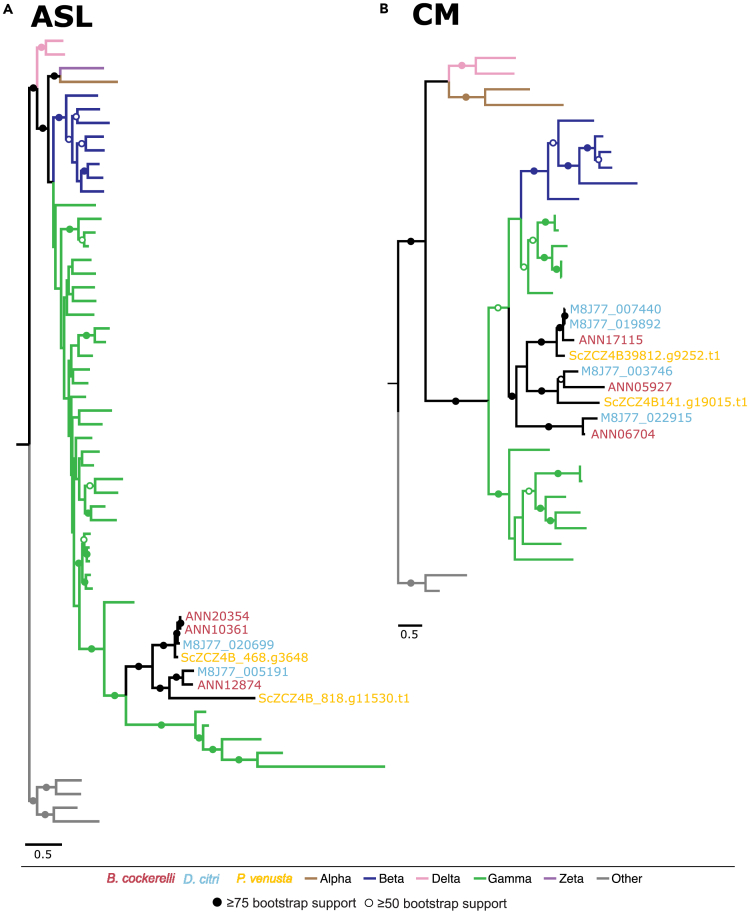
Phylogenetic analyses of gene duplications for HTGs in the psyllid genomes of *P. venusta* (GenBank: GCA_012654025.1), *D. citri* (GenBank: GCA_024506315.2), and *B. cockerelli* (GenBank: GCA_024516035.1) for (A) argininosuccinate lyase (*ASL*) genes and (B) chorismate mutase (*CM*) genes Branches are colored according to their bacterial classes within the Proteobacteria, and ranges of bootstrap values are indicated by filled or open circles as indicated in the key.

Expression levels of HTGs were examined for differential gene expression in bacteriomes compared to body for both psyllid species *B. cockerelli* and *D. citri* (Table 2). Two of these HTGs are predicted to collaborate with *Carsonella* for the biosynthesis of the essential amino acids, arginine and phenylalanine.^7^^,^^22^ These genes include argininosuccinate lyase (*ASL*, EC 4.3.2.1), which catalyzes the terminal step in the arginine biosynthesis pathway, and chorismate mutase (*CM*, EC 5.4.99.5), which is responsible for the conversion of chorismate into an intermediate precursor in the phenylalanine pathway (Figure 2). Phylogenetic analyses carried out in this study reveal distinct patterns within the genomes of *B. cockerelli* and *D. citri*. Specifically, we observed the presence of three distinct gene copies of *ASL* and *CM* in the genome of *B. cockerelli*, whereas *D. citri*’s genome possesses two gene copies of *ASL* and four gene copies of *CM* (Figure 3). All copies of *ASL* and *CM* in both psyllid species were significantly up-regulated in bacteriomes compared to the body tissues (Table 2; Figure 3), suggesting that all gene copies play important roles in mediating the symbiosis with *Carsonella* for arginine and phenylalanine biosynthesis.

**Table 2:** Differential gene expression for HTGs in bacteriomes compared to the body tissues in *B. cockerelli* and *D. citri*

	*B. cockerelli*^a^	LogFC	FDR	*D. citri*	LogFC	FDR
**Argininosuccinate lyase**						

*ASL*-1	ANN12874	3.75	4.81E-40	M8J77_005191	3.27	2.13E-29
*ASL*-2a	ANN10361	7.63	7.48E-93	M8J77_020699	10.07	7.17E-93
*ASL*-2b	ANN20354	8.06	4.11E-11			

**Chorismate mutase**						

*CM*-1	ANN05927	2.63	4.89E-11	M8J77_003746	4.26	3.79E-65
*CM*-2	ANN06704	3.07	4.02E-41	M8J77_022915	3.98	3.37E-45
*CM*-3	ANN17115	7.403	5.66E-40	M8J77_007440	8.84	4.20E-96
				M8J77_019892	8.84	4.20E-96

**A/G-specific adenine glycosylase**						

*MUTY*	ANN05978	−2.66	1.66E-85	M8J77_002314	−2.19	3.37E-05

**AAA-ATPase-like**						

*ORF*-1	ANN01458	6.99	2.07E-09	M8J77_017214	5.85	3.62E-66
*ORF*-2	ANN17655	6.86	5.70E-38	M8J77_013596	7.96	5.46E-73
*ORF-*3a	ANN18155	6.957	7.57E-42	M8J77_011284	6.90	1.64E-75
*ORF*-3b	ANN18147	11.68	1.60E-113	N/A	N/A	N/A

**Riboflavin synthase**						

*RIBC*	ANN14810	7.88	1.07E-83	M8J77_002167	6.15	3.21E-85

**16S rRNA methyltransferase**						

*RSMJ*	ANN13802	11.70	1.44E-86	M8J77_006534	5.87	3.27E-46

**VOC family protein**						

*YDCJ*	ANN12599^a^	0.28	0.23	M8J77_004361	1.55	8.64E-10

Homologs in the same row are one-to-one or one to many orthologs. “N/A” indicates no ortholog was identified.

a All genes in the table are significantly differentially expressed except for ANN12599.

For the other HTGs in the psyllid genomes, six out of eight HTGs share the same expression pattern for both *D. citri* and *B. cockerelli.* These HTGs exhibit significant up-regulation in the bacteriomes compared to the body tissues, indicating their involvement in vital cellular processes. For instance, five HTGs were significantly up-regulated in bacteriomes compared to the body tissues (*RIBC*, *RSMJ*, ORF-1, -2, and -3a) for both psyllid species and are predicted to be involved in riboflavin biosynthesis (*RIBC*), rRNA methylation (*RSMJ)* and diverse cellular activities (ORFs, AAA-ATPase). One ortholog was significantly down-regulated (*MUTY)* in bacteriomes compared to body tissues for both psyllid species (Table 2) and is predicted to encode an A/G specific mismatch repair enzyme. Gene expression patterns here indicate that this latter enzyme may play a more important role in body tissues compared to the bacteriome for both psyllid species (Table 2). The HTG *YDCJ*, a conserved bacterial gene which belongs to the vicinal oxygen chelate (VOC) family, showed species-specific expression where *YDCJ* was significantly only up-regulated in *D. citri* bacteriomes (Table 2).

### Lineage-specific gene clusters differentially expressed in bacteriomes

To explore the evolutionary divergence and functional specialization of bacteriome expression in *D. citri* and *B. cockerelli*, we investigated lineage-specific gene clusters in both psyllid species. These gene clusters are unique to either *D. citri* or *B. cockerelli*, encompassing a total of 1,314 clusters comprising 5,150 genes in *D. citri* and 724 clusters comprising 3,133 genes in *B. cockerelli* (Table S6). In *D. citri*, 26 percent of lineage-specific genes (1,331 genes) were significantly differentially expressed, where a total of 525 and 804 genes were significantly up- and down-regulated, respectively, in bacteriomes compared to the body tissues (Table S7). The up-regulated lineage-specific genes in *D. citri* are primarily associated with nucleus-related activities, transposition and the regulation of the Notch signaling pathway (Table S7). The down-regulated lineage-specific genes in *D. citri* are primarily associated with DNA integration, RNA-directed DNA polymerase activity and the tachykinin receptor signaling pathway (Table S7).

In *B. cockerelli*, 24 percent of lineage specific genes (749 genes) were significantly differentially expressed, where a total of 427 and 322 genes were significantly up- and down-regulated, respectively, compared to the body tissues (Table S8). The up-regulated lineage-specific genes in *B. cockerelli* are primarily associated with protein processing, telomeric D loop disassembly, and transposition (Table S8). The down-regulated lineage-specific genes in *B. cockerelli* are primarily associated with locomotor rhythm, protein dephosphorylation, positive regulation of transcription, and proteolysis (Table S8).

Additionally, we examined the gene expression patterns of significantly expanding gene families previously identified in *B. cockerelli*.^22^ Out of 3,375 significantly expanding genes in 157 clusters, we observed that almost one-third of genes (a total of 1,011 genes) were significantly differentially expressed in bacteriomes relative to the body tissues in *B. cockerelli* (Table S9). Over 60 percent of these genes in 119 clusters were significantly up-regulated in bacteriomes compared to the body tissues, and the remaining genes in 99 clusters was significantly down-regulated in bacteriomes compared to the body tissues (Table S9). The up-regulated genes are primarily associated with transposons and gene regulation, such as nucleic acid and ATP binding proteins (Table S9A).

### KEGG pathway analyses

**Figure 4:**
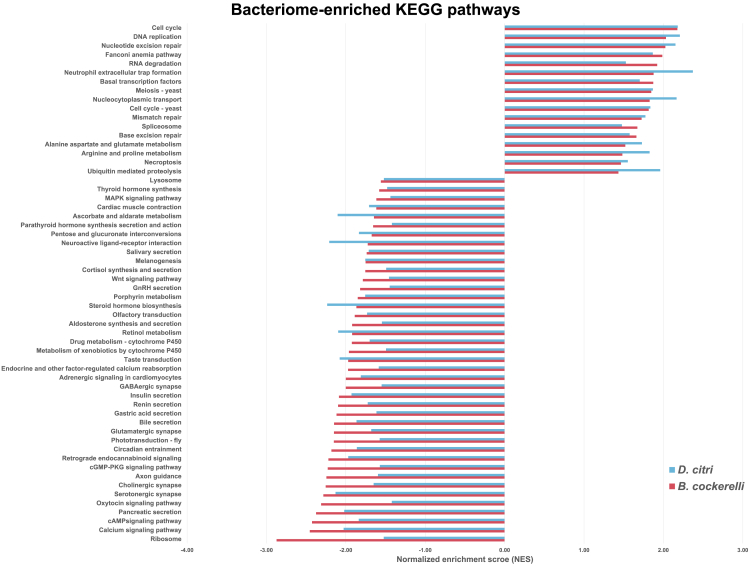
Gene Set Enrichment Analyses of *D. citri* and *B. cockerelli* displaying all KEGG pathways that were significantly enriched in bacteriomes compared to the body tissues based on transcriptomics data Significance = the normalized p ≤ 0.05 and FDR q ≤ 0.25.

Using GSEA with KEGG pathways, we investigated the enrichment of biological pathways in the bacteriomes compared to body tissues for both species. In the bacteriomes of both psyllid species, we identified 17 and 39 pathways that were significantly positively and negatively enriched in bacteriomes compared to the body tissues, respectively (Figure 4; Table S10). The significantly positively enriched pathways in the bacteriomes include processes such as cell growth and death, transcription, nucleic acid replication and repair, and amino acid metabolisms (Figure 4; Table S10). The positive enrichment of these latter pathways in bacteriomes suggests that bacteriomes are metabolically highly active compared to other body cells, especially within the amino acid metabolism. The significantly negatively enriched pathways in bacteriomes were primarily involved in signaling pathways, lysosomes, and the digestive and endocrine systems (Figure 4; Table S10). These results indicate that genes involved in cell communication, and hormone and enzyme production, such as in the digestive and endocrine systems, are turned off and/or dampened in expression in the bacteriome. Furthermore, each psyllid species also exhibits species-specific pathways that were either positively or negatively enriched in their bacteriomes. For example, 45 and four unique KEGG pathways were significantly positively enriched in *D. citri* and *B. cockerelli* bacteriomes compared to the body tissues, respectively, and eight and 60 unique KEGG pathways were significantly negatively enriched in *D. citri* and *B. cockerelli* bacteriomes compared to the body tissues, respectively (Table S10). For example, in *D. citri* the sulfur metabolism and vitamin pathways related to B6 and folate were only significantly positively enriched in *D. citri* bacteriomes. In *B. cockerelli* the N-glycan biosynthesis pathways were only significantly positively enriched in *B. cockerelli* bacteriomes (Table S10). These results suggest that bacteriomes may play species-specific roles in the expression of pathways that are involved in the biosynthesis of sulfur, vitamins, and the modulation of protein folding, stability, and trafficking in *D. citri* compared to *B. cockerelli.*

### Tissue-specific quantification of *Wolbachia* in *D. citri* and *B. cockerelli*

**Figure 5:**
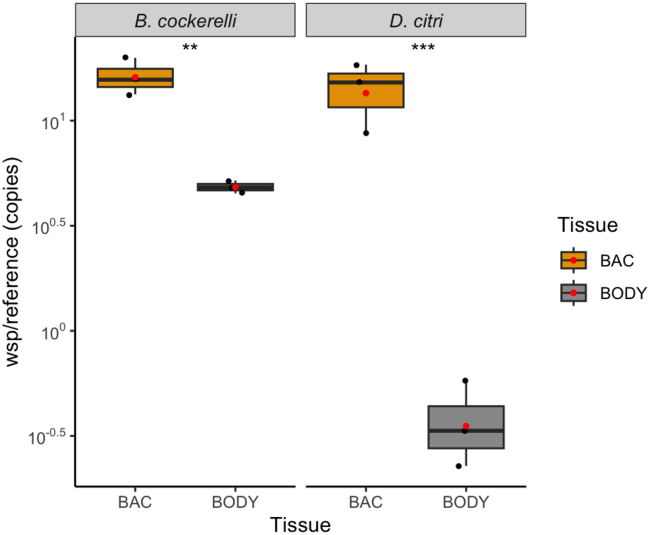
Boxplots showing the *Wolbachia wsp* gene copy numbers in bacteriomes and body tissues for *B. cockerelli* and *D. citri* The *wsp* gene copy numbers were normalized to the psyllid single copy house-keeping genes encoding *FORKHEAD* and *ACTB* in *B. cockerelli* and *D. citri*, respectively. All data points of three biological replicates of each tissue type for both psyllid species are presented. Tissues for each biological replicate are pooled tissues from 15 fifth instar nymphs. Red dots and asterisks represent means and significance (p < 0.01 ∗∗, p < 0.001 ∗∗∗) of normalized abundance values using a t-test.

We quantified genome copy numbers of the *Wolbachia* endosymbiont from both psyllid species in the bacteriome and body tissues to assess how the *Wolbachia* chromosomal copies fluctuate depending on the tissue type and whether it may directly interact with the integrative metabolism inside of the bacteriome. For both psyllid species, *Wolbachia* genome copies relative to psyllid genome copies were significantly higher in bacteriomes compared to body tissues (Figure 5). The copy number of the single copy *Wolbachia* gene (*wsp)* in *B. cockerelli* relative to the single copy psyllid gene (*FORKHEAD*) was 16X ± 3 (mean ± standard deviation, n = 3) higher in bacteriomes and 5X ± 0.3 (n = 3) higher for body tissues (Figure 5). In *D. citri*, the copy number of the single copy *Wolbachia* gene (wsp) gene relative to the single copy psyllid gene (*ACTB*) was 14X ± 5 (mean ± standard deviation, n = 3) higher for bacteriomes and 0.4X lower ±0.2 (n = 3) for body tissues (Figure 5). These findings indicate a potential direct interaction between *Wolbachia* and both psyllid hosts and *Carsonella* (and *Profftella* in *D. citri*) within the bacteriome, suggesting that *Wolbachia* could have a substantial impact on the integrated metabolism between *Carsonella* and the psyllid host.

## Discussion

### Conservation and divergence of gene expression patterns between two psyllid species for the integrated metabolism of Psylloidea and *Carsonella*

In this study, we observed both conserved and variable gene expression profiles for specialized hemipteran cells (bacteriome) that harbor the nutritional endosymbiont *Carsonella* for two psyllid species that diverged ∼86 million years ago^22^ (Figures 1 and 2; Table 1). Drawing on previous transcriptomic and proteomic research conducted in Hemiptera,^2^^,^^6^^,^^7^^,^^8^^,^^19^^,^^36^^,^^37^^,^^38^ we specifically investigated homologs that are postulated to collaborate with the obligate nutritional endosymbiont, *Carsonella*. Five out of nine enzymes (55%) that are predicted to collaborate in six of *Carsonella*’s essential amino acid pathways (arginine, isoleucine, valine, leucine, phenylalanine, and methionine) were significantly up-regulated in psyllid bacteriomes compared to body tissues for both *B. cockerelli* and *D. citri* (Figure 2). Interestingly, there is a high level of conservation for the differential expression patterns of these collaborative genes in bacteriomes when compared to the more distantly related psyllid species*, P. venusta*.^7^ In both *P. venusta and D. citri*, as well as *P. venusta* and *B. cockerelli,* a notable 75% and 62% of these collaborative genes, respectively, exhibited significant up-regulation in bacteriomes compared to body tissues (Figure 2).

### Species-specific control of amino acid biosynthesis and its relation to psyllid microbiome differences

Even though the majority of the psyllid’s collaborative genes displayed similar expression patterns in bacteriomes, it is noteworthy that differences in expression patterns were observed between species. Some of these species-specific differences may be attributed to microbiome differences between the psyllid species. For example, a key difference between *D. citri* and *B. cockerelli’s* microbiomes is the presence of a co-endosymbiont, *Profftella*, in *D. citri.* Here we found that the sulfur metabolism and the cysteine and methionine metabolism were only significantly positively enriched in *D. citri* bacteriomes, suggesting that *Profftella* may play a key role in collaborating with *D. citri* for the biosynthesis of sulfur-containing metabolites (Table S10). Indeed, in *D. citri,* the enzymatic machinery required for sulfate reduction is exclusively found in *Profftella* (*cysDHIN*).^24^ In contrast *Carsonella* has completely lost all genes associated with this pathway, and there is no evidence suggesting that these genes have been substituted by any functionally acquired HTGs in the psyllid host.^7^^,^^22^ In the model aphid-*Buchnera* system, the sulfur assimilation pathway is generally present in *Buchnera*, and it is primarily inferred to have a role in synthesizing cysteine from serine.^39^ The origin of cysteine for *D. citri* remains uncertain as the absence of *cysEK* in *Carsonella* hinders the completion of the pathway, making it unclear whether cysteine is synthesized from serine.^22^ A previous hypothesis put forth in *P. venusta*^7^ suggests that cysteine could potentially be synthesized from cystathionine through the activity of cystathionine gamma-lyase (*CGL*). It is plausible that a similar mechanism may also operate in *D. citri.* Future studies will be needed to determine the exact role of sulfur assimilation in *Profftella* and its interactions with *D. citri*.

Another important species-specific expression difference for collaborative psyllid genes was observed for the enzyme *BCAT*, which serves as the final step in the biosynthesis of the branched-chain amino acids, isoleucine, valine, and leucine. The expression pattern of the collaborative enzyme *BCAT* varied among the three divergent psyllid species, where it was up-regulated in bacteriomes in *D. citri*, down-regulated in *B. cockerelli*, and non-significantly differentially expressed in *P. venusta* (Figure 2). One possible explanation for these divergent expression patterns is that, unlike *Buchnera* in aphids^2^ and *Tremblaya* and *Moranella* in mealybugs,^6^ all *Carsonella* genomes still encode *ilvE,* which potentially has a similar enzyme function as *BCAT* in these latter insect bacteriomes. Therefore, *BCAT* may not be essential in some psyllid bacteriomes for this collaborative, redundant function. In *D. citri*, both *Profftella* and *Wolbachia* lack the *ilvE* gene in their genomes (Table S11).^24^^,^^40^ Therefore, the observed up-regulation of *BCAT* in *D. citri*, as compared to *B. cockerelli* and *P. venusta*, could potentially be attributed to the presence of *Profftella* and *Wolbachia* in *D. citri’s* bacteriomes. This presence might lead to an increased nutritional demand for essential amino acids, such as leucine, valine, and threonine, consequently relying more on the psyllid host for the regulation of these branched-chain amino acids.

In this study both psyllid species harbor *Wolbachia,* which is in contrast to the previous analysis of *P. venusta*.^7^ *Wolbachia* is currently categorized in 16–17 distinct monophyletic lineages known as “supergroups” from A to S,^41^ where supergroups A and B are found only in arthropods.^42^ The *Wolbachia* strains discovered in psyllid species to date all belong to supergroup B.^43^^,^^44^ Due to its limited capacity for amino acid biosynthesis, *Wolbachia* supergroups A and B have been traditionally known to be highly dependent on the amino acid metabolism of its hosts,^45^^,^^46^ while recent studies have provided evidence of evolutionary transitions from facultative to obligate mutualisms in certain members of the *Wolbachia* F supergroups.^47^^,^^48^ Here higher genome copy counts of *Wolbachia* were measured in *D. citri* and *B. cockerelli* bacteriomes compared to body tissues (Figure 5), suggesting that *Wolbachia* may have direct metabolic influences on the host and obligate symbiont(s) within bacteriomes for amino acids. A study by Ren et al. (2018)^49^ corroborates our data here and found that *Wolbachia*-DC was detected with the highest density in the *D. citri* bacteriome compared to other body tissues using Fluorescence *In Situ* Hybridization (FISH) visualization. Hosseinzadeh et al. (2019),^50^ however, found that *Wolbachia*-DC was the most abundant in *D. citri*’s Malpighian tubules among a variety of organs including heads, guts, testes/ovaries and bacteriomes. To our knowledge, it is not known whether *Wolbachia* has any role in osmoregulation or nitrogen excretion, which are the primary functions of the Malpighian tubules. Based on *Wolbachia*-DC’s genome, it does not have the enzymatic capabilities required for recycling nitrogen from uric acid,^40^ which can be found in other insect bacterial endosymbionts.^51^^,^^52^^,^^53^

While *Wolbachia* exhibits genomic hallmarks of being parasitic and reliant on amino acids from its psyllid host and *Carsonella*, it may also play a role in essential amino acid biosynthesis and exhibit facultative or even mutualistic interactions. For example, cysteine-S-conjugate beta-lyase (*CBL*; EC 4.4.1.13), is encoded in both *D. citri* and *B. cockerelli* and is hypothesized to collaborate with *Carsonella* in the methionine biosynthesis pathway.^7^ Interestingly, the two copies of *CBL* in *D. citri* exhibited significant up-regulation in bacteriomes, while the *CBL* copies in *B. cockerelli* showed no differential regulation in bacteriomes (Table 1). The bacterial enzyme *metC* (EC 4.4.1.13), a cystathionine β-lyase enzyme in bacteria, is widely conserved among different *Wolbachia* strains (Table S11)^47^^,^^54^ suggesting that *Carsonella* might not be entirely dependent on host-encoded *CBL* for methionine biosynthesis. Instead *Wolbachia* may help complement the methionine biosynthesis pathway in *B. cockerelli*. Further functional genomics analyses of *Wolbachia* in *B. cockerelli* are needed to further understand this potential metabolic interaction.

### Role of the glutamate dehydrogenase pathway in the recycling of waste ammonia in bacteriomes

Sap-feeding insects feed on a diet that is limited in nitrogen, especially essential amino acids.^55^^,^^56^ The recycling of waste ammonia in sap-feeding mutualistic relationships serves as a potential solution to fuel the nitrogen-demanding nature of these symbioses. One enzyme that can upgrade nitrogen from compounds such as ammonia is *GS*, which is significantly up-regulated in bacteriomes of both *D. citri* and *B. cockerelli* here. The *GOGAT* gene, which works in a cycle with *GS* to produce glutamate however is down-regulated in *B. cockerelli* and not statistically differentially expressed in *D. citri* (Figure 1; Table 1). The GS/GOGAT cycle is known to be an important mechanism in symbiotic relationships between sap-feeding insects and their obligate nutritional symbionts, as highlighted by previous studies on the upgrading of nitrogen from waste ammonia to fuel the amino acid metabolism.^2^^,^^6^^,^^7^^,^^19^^,^^38^^,^^57^^,^^58^ Interestingly, we identified that glutamate dehydrogenase (*GDH*) was significantly up-regulated in both *D. citri* and *B. cockerelli* bacteriomes, which might be an alternative to *GOGAT* for recycling ammonia into glutamate (Figure 2). The enzyme *GDH* is known to play a key role in assimilating waste nitrogen for the production of amino acids in human breast cancer cells where ammonia accumulates rapidly.^59^ Another recent study also revealed that raised levels of ammonia (hyperammonemia) results in *GDH* to play an essential role in the recycling of ammonia within non-cancerous brain cells.^60^ As a result, when ammonia levels rise in bacteriomes, the *GS/GOGAT* cycle may undergo a shift in its role, favoring *GDH* for the recycling of ammonia in the biosynthesis of amino acids.

Most studies on hemipteran systems have not reported any involvement of the GDH pathway.^2^^,^^6^^,^^7^^,^^38^ However, a recent study has found a potential role of the GDH pathway in the glassy-winged sharpshooter (GWSS) symbiosis system.^37^ In a novel dual obligate symbiosis between *Baumannia* and *Sulcia*-GWSS, where the GWSS host has two types of bacteriome tissues for the two endosymbionts, the *GDH* gene of GWSS is more highly expressed than *GOGAT* of the GWSS in the yellow bacteriome that harbors both *Baumannia* and *Sulcia*-GWSS.^37^ Future studies are warranted to investigate whether the up-regulation of *GDH* or the GS/GOGAT cycle in insects for nitrogen upgrading in bacteriomes for amino acid biosynthesis is influenced by the symbiosis, microbiome composition, and/or environmental conditions.

### Functional significance of horizontally transferred genes in the amino acid metabolism

In sternorrhynchan insects, a consistent pattern arises with the loss of endosymbiont enzymes involved in the terminal steps of essential amino acid biosynthesis pathways. In response, host genes are hypothesized to step in and bridge these gaps.^39^ Notably, this pattern extends to the two HTGs in the psyllid’s genome, *ASL* providing the terminal step for the arginine pathway and *CM* providing the terminal step for the phenylalanine pathway in psyllids. Here we found the significant up-regulation of these HTGs in bacteriomes for both psyllid species (Figures 2 and 3). This finding aligns with previous research conducted in *P. venusta*, suggesting a consistent pattern of HTG up-regulation in psyllid bacteriomes when compared to body tissues.^7^ Despite the potential for subfunctionalization and/or neofunctionalization of the paralogous copies of *ASL* and *CM*, all of the copies were significantly up-regulated in the bacteriomes compared to the body tissues in both psyllid species *B. cockerelli* and *D. citri* (Table S4.2). This suggests the potential importance of gene dosage for these collaborative enzymes within bacteriomes. Further investigation is needed to determine whether these gene copies exhibit differential expression in response to varying environmental conditions.

This study also identified the HTG *RSMJ,* a bacterial 16S rRNA methylation gene to have one of the highest relative expression levels of any gene in the bacteriome compared to the body for both psyllid species, as determined by comparative transcriptomics analysis using PCA (Tables 2 and S5). Phylogenetic analysis revealed that the *RSMJ* gene from the three psyllid species cluster within the ‘class I SAM-dependent methyltransferase’ group within Gammaproteobacteria (e.g., *Escherichia coli, Photobacterium* spp.)^61^ (Figure S3). The InterPro GO term (GO:0008990) and Superfamily annotations (IPR029063) provide further evidence for classifying psyllid *RSMJ* as a SAM-dependent 16S rRNA methyltransferase, however the precise role of this gene in psyllid bacteriomes and the factors contributing to its conserved high expression levels in psyllids remain uncertain without additional functional assays. In *E. coli*, ten 16S rRNA methylation genes are involved in the modification of the small ribosomal subunit. The gene *rsmJ* in *E. coli* is characterized as a methyltransferase specific for the methylation of guanosine in the 1516 position of the 16S rRNA that has a cold sensitive mutant phenotype.^62^ In bacteria, rRNA methylation serves various purposes, including facilitating rRNA development, enhancing the stability of rRNA configurations, and modifying translation speed, however, alternative roles have been described, such as conferring resistance against aminoglycoside antibiotics derived from actinomycetes.^63^

Horizontal gene transfer events in psyllids are also hypothesized to play a critical role in B vitamin biosynthesis. Insects require eight B vitamins as coenzymes for essential reactions, but they are unable to produce these vitamins and must obtain them through their diet.^64^ Some insects can supplement B vitamins using their microbial symbionts,^39^^,^^65^ while some genes in vitamin biosynthesis have been acquired from bacteria by the insect host.^6^^,^^7^ In psyllids, the *RIBC* gene, also known as riboflavin synthase, has been found to be horizontally transferred to the host genome.^7^^,^^22^ This gene in bacteria, *ribC,* catalyzes the final step of the pathway, converting 6,7-dimethyl-8-ribityllumazine (DMRL) into riboflavin.^66^ Our data shows that *RIBC* was significantly up-regulated in bacteriomes at very high levels in both *B. cockerelli* and *D. citri* (LogFC = 7.88 and 6.15, respectively), similar to *RIBC* in *P. venusta*.^7^ However, only *D. citri* has been reported to possess a complete riboflavin biosynthetic pathway, where the co-obligate endosymbiont *Profftella* in *D. citri* retains all the genes in the riboflavin biosynthesis pathway except for the *ribC* (Figure 2).^24^ In contrast, the genomes of both the host and *Carsonella* in *B. cockerelli* and *P. venusta* appear to lack the rest of the genes in the riboflavin biosynthesis pathway. Therefore *B. cockerelli* and *P. venusta* may rely on their diet for the acquisition of the intermediate DMRL to produce riboflavin.

### Regulation of lineage-specific gene clusters and its implications in evolutionary divergence

Our analysis of lineage-specific gene clusters that are unique to either *D. citri* or *B. cockerelli* highlights gene clusters that are differentially expressed in bacteriomes compared to body tissues for each species. Notably, the up-regulation of the Notch signaling pathway was prominent among *D. citri*-specific gene clusters, while no *B. cockerelli*-specific gene clusters were related to this pathway (Tables S6 and S7). The Notch signaling pathway, particularly in insects, plays crucial roles in body segmentation, proliferation, embryogenesis and cell fate determination.^67^ In the aphid-*Buchnera* system, it has been observed that the signaling pathways, such as TGF-beta, Wnt, Hippo, Hedgehog and Notch, are significantly enriched in the 3^rd^ instar bacteriocytes^58^ and the 4^th^ instar bacteriocytes of low-*Buchnera* titer genotypes.^68^ Smith and Moran (2020)^68^ hypothesize that the up-regulation of signaling pathways such as Notch in bacteriomes may result in an increase in both the number and size of aphid bacteriocytes in response to essential amino acid limitation.^69^ Hence, the observed up-regulation of lineage specific genes associated with the Notch signaling pathway in *D. citri* suggests that *D. citri* may possess distinct developmental processes that necessitate precise regulation to maintain a balance between metabolic production and cell growth. This phenomenon could be attributed to the heightened nutritional requirements imposed by *Profftella*, which is absent in *B. cockerelli*.

Our analysis showed that telomeric D loop disassembly was one of the most represented up-regulated GO-terms for *B. cockerelli* lineage specific clusters, and this GO-term was not found in *D. citri* specific gene clusters. (Tables S7 and S8). Telomeric D loop disassembly is a critical process involved in chromosome stability, integrity, and maintenance.^70^ The telomere motif (TTAGG)n is considered canonical for insects,^71^^,^^72^ however, the structure of telomeric repeats can vary depending on the insect group as observed in some coleopteran species.^73^^,^^74^ Interestingly, in a distantly related hemipteran species *Myzus persicae*, the (TTAGG)n motif has been found to be interspersed with inserted non-LTR retrotransposable elements,^75^ which could potentially be an intermediate state between the canonical insect telomere and retrotransposon-based ones.^76^ It is not clear at this point whether *B. cockerelli* has a canonical or retrotransposon-based (or intermediate) telomeric structure. To date the telomere structure in psyllids has been investigated for only five species within four genera from the families Psyllidae and Aphalaridae,^77^ and all five species in the study appear to have the (TTAGG)n motif according to FISH visualization. Nevertheless, given that transposition is another highly represented GO-term for *B. cockerelli* specific gene clusters in bacteriomes, and that *B. cockerelli* shows a significant increase of transposable elements in its genome,^22^ the up-regulation of lineage specific genes related to the telomeric D loop assembly in *B. cockerelli* bacteriomes suggests that there might be a potential association between telomere maintenance and transposable elements that differs from *D. citri* bacteriomes.

### Conclusion

This study found both conserved and variable gene expression profiles for bacteriomes of two divergent psyllid species. Both psyllid species rely on *Carsonella* for the production of essential amino acids however their regulation of this symbiosis may vary due to host and symbiont genetic factors, differences within their microbiomes, and/or the environment including diet. Future studies aiming to investigate additional psyllid species, as well as considering various factors within species, will provide valuable insights into the evolutionary mechanisms underlying the integrated metabolisms of psyllids. This comprehensive approach will contribute to a deeper understanding of the complex dynamics involved in the evolution of metabolic interactions in psyllids.

### Limitations of the study

Functional genetics techniques are still not available in these two psyllid species therefore metabolomic analyses and interpretations here are based on transcriptome data and comparative evolutionary genomics analyses. Due to available insectary and quarantine space, available funding, and timing of life stage dissections of this project, which were conducted around the same time frame to prevent batch effects, only one psyllid genetic line was used per species for this study.

## STAR★Methods

### Key resources table

**Table:** 

REAGENT or RESOURCE	SOURCE	IDENTIFIER
**Bacterial and virus strains**		

*Escherichia coli* K12 JM109	Promega	Cat#L2005

**Biological samples**		

*Diaphorina citri* bacteriome tissues	This study	N/A
*D. citri* body tissues	This study	N/A
*Bactericera cockerelli* bacteriome tissues	This study	N/A
*B. cockerelli* body tissues	This study	N/A

**Deposited data**		

BioProject	This study	NCBI BioProject: PRJNA939696
Raw data	This study	NCBI SRA: SRR23641431 to SRR23641440

**Experimental models: Organisms/strains**		

*D. citri*	USA:CA	N/A
*B. cockerelli*	USA:CA	N/A

**Oligonucleotides**		

Dcit-wsp-qF/R	This study	See STAR methods section for sequences
Bcoc-wsp-qF/R	This study	See STAR methods section for sequences

**Recombinant DNA**		

pGEM-T-Easy-Dcit-wsp	This study	N/A
pGEM-T-Easy-Bcoc-wsp	This study	N/A
pGEM-T-Easy-ACTB	This study	N/A
pGEM-T-Easy-FORKHEAD	This study	N/A

**Software and algorithms**		

FASTQC	Andrews (2010)^81^	v.0.11.8
Trimmomatic	Bolger et al. (2014)^82^	v.0.36
HiSAT	Kim et al. (2015)	v.2.1.0
StringTie	Pertea et al. (2015)^84^	v.2.2.1
RStudio	R Core Team (2022)^85^	v.4.2.0
EdgeR	Robinson et al. (2010)^86^	N/A
BlastKOALA	Kanehisha et al. (2016)	N/A
OrthoVenn2	Xu et al. (2019)^92^	N/A
GSEA	Subramanian et al. (2005)^88^	v4.0.0
RAxML-HPC BlackBox	Miller et al. (2010)^90^	v.8.2.12
Figtree	Rambaut (2018)^91^	v.1.4.4
ggplot2	Wickham (2016)^98^	N/A

**Other**		

*D. citri* reference genome	Carlson et al. (2022)^21^	GCA_024506315.2
*B. cockerelli* reference genome	Kwak et al. (2023)^22^	GCA_024516035.1

### Resource availability

#### Lead contact

Further information and requests for resources can be directed to and will be fulfilled by the lead contact, Allison K. Hansen (allison.hansen@ucr.edu).

#### Materials availability

This study did not generate new unique reagents.

### Experimental models and study participants

#### Plant and insect materials

The *B. cockerelli* line was derived from a wild population in Temecula, California, USA, in August 2019, and is from the same culture that was sequenced in Kwak et al. (2023).^22^ The established line was maintained on 8-12-week-old *Capsicum annuum* plants (California Wonder pepper) at 25°C under a 16L:8D light/dark cycle. The *Diaphorina citri* nymph culture was obtained from the Stouthamer lab at the University of California (UC), Riverside, and was maintained on 6-month-old *Murraya koenigii* leaves (Curry tree) at 27°C under a 16L:8D light/dark cycle.

For both *B. cockerelli* and *D. citri*, 60 bacteriomes and the rest of the body, without the bacteriomes, were dissected from 5^th^ instar nymphs and pooled into two separate tissue samples similar to Hansen & Moran (2011),^2^ resulting in three biological replicates per species (N = 12 samples). The *D. citri* and *B. cockerelli* nymphs were aged for bacteriome dissections according to their morphological characters and developmental periods as described in Hall et al. (2013)^79^ and Knowlton & Janes (1931),^80^ respectively. Samples were stored at −80°C in the RNAprotect bacterial reagent (QIAGEN, Germantown, MD, USA) until RNA extraction.

### Method details

#### Total RNA sample preparation and RNA sequencing

Total RNA was purified, DNase 1 treated, and cleaned using Quick-RNA Microprep kit and RNA Clean & Concentrator kit-5 (Zymo research, Irvine, CA, USA) following the manufacturer’s instructions. Purified RNA sample quality and quantity were measured using the Bioanalyzer 2100 (Agilent, Santa Clara, CA, USA) and Qubit 4.0 Fluorometer (Invitrogen, Carlsbad, CA, USA) at the Institute of Integrative Genome Biology Instrumentation Facilities Services at the University of California, Riverside.

High-quality total RNA (>1 μg) from each pooled bacteriome sample and corresponding body tissue sample was submitted to the DNA Technology Core at the University of California, Davis for library preparation and sequencing. Strand-specific and barcode indexed RNA-seq libraries were generated from 300ng total RNA for each sample. Poly-A enrichment and library prep was done using the Kapa mRNA Stranded library preparation kit (KK8421, Kapa Biosystems, Cape Town, South Africa), following the instructions of the manufacturer. Libraries were amplified with 12 cycles of PCR. The fragment size distribution of the libraries was verified via micro-capillary gel electrophoresis on a Bioanalyzer 2100 (Agilent, Santa Clara, CA). The libraries were quantified by fluorometry on a Qubit fluorometer (LifeTechnologies, Carlsbad, CA) and pooled in equimolar ratios. The pool was quantified by qPCR with a Kapa Library Quant kit (Kapa Biosystems) and sequenced on 1 lane of the Illumina NovaSeq S4 platform (Illumina, San Diego, CA) with paired-end 150bp reads. Reads for all RNA-Seq samples were submitted to the Sequence Read Archive of the National Center for Biotechnology Information (NCBI) under BioProject ID PRJNA939696.

#### Ortholog analysis and interspecies comparative transcriptomic analysis

Orthologous clusters, lineage-specific clusters, and one-to-one orthologs of *D. citri* and *B. cockerelli* were determined using OrthoVenn2^92^ using default settings. To visualize the differentially expressed one-to-one orthologs, a Venn diagram was generated using SeqCode VennPlotter.^93^ Similar to Georgiadou et al. (2022)^33^ and Argandona et al. (2023),^32^ one-to-one orthologs were further examined for inter-species gene expression comparisons. Specifically, the relative magnitude of changes of orthologs in bacteriomes were compared to body tissues for each species using Principal Components Analyses (PCA). To do this, we selected one-to-one orthologs that displayed significant differential gene expression (see above for the thresholds) in both *D. citri* and *B. cockerelli*. These orthologs were ranked in descending order of absolute logFC, and each ortholog was then assigned a value of 100 divided by rank, which was then multiplied by the sign of the original logFC. These values were then used as the input for PCA to identify one-to-one orthologs that display the most similar and different gene expression profiles in the bacteriomes compared to the body tissues. The PCA was performed using PC-ORD (version 4.25) ^94^. For each principal component (axis 1 and 2), we selected the top 50 genes with the highest correlations (the top 25 genes for positive or negative correlations) from the principal components output loading matrix similar to Korb et al. (2021).^34^ We examined the GO annotations for these top 100 orthologs using OrthoVenn2.^92^ We generated a heatmap of logFC for one-to-one orthologs using SeqCode Heatmapper.^93^

#### Quantitative real-time PCR

Fifth instar nymphs of both species were collected for bacteriome dissections for *Wolbachia* endosymbiont quantification similar to the RNAseq analysis (above). Bacteriomes and the rest of the body tissues without the bacteriomes were pooled from 15 individuals of both sexes for each biological replicate with a total of three biological replicates per species per tissue type. Genomic DNA was extracted from pooled bacteriome and body tissues using Quick-DNA Microprep Plus Kit (Zymo, Irvine, CA, USA). Samples were homogenized, lysed, and purified following the Solid Tissue Protocol. DNA samples were treated with RNase A (Thermo Scientific, Waltham, MA, USA), and cleaned with the Genomic DNA Clean and Concentrator kit-10 (Zymo research, Irvine, CA, USA). Purified DNA sample quality and quantity were measured via QuickDrop (Molecular Devices, San Jose, CA, USA) and Qubit 4.0 Fluorometer (Invitrogen, Carlsbad, CA, USA).

Primers for the *Wolbachia* surface protein (*wsp*) gene were designed for this analysis using Primer-BLAST in NCBI using GenBank: MN809922.1 for *D. citri* (Dcit-wsp-qF/R: 5′-TGCTGGAGCTCGTTACTTCG-3’/5′- CAGCTTCTGCACCAACAGTG-3′) and GenBank: KM267307.1 for *B. cockerelli* (Bcoc-wsp-qF/R: 5′- ATAGCTGCTGGTGGTGCATT-3’/5′- CACCAACACCAACACCAACG-3′). For calibration, the psyllid housekeeping genes encoding *ACTB*^95^ and *FORKHEAD*,^96^ which are single-copy genes in the *D. citri and B. cockerelli* genomes, respectively, were also quantified. The PCR products were cloned into the pGEM-T Easy vector (Promega) and amplified in *Escherichia coli* JM109. Plasmids with inserts were amplified in *E. coli* and purified using Plasmid Minikit (Invitrogen). Subsequently, Plasmid DNA was quantified using a Qubit 4.0 Fluorometer (Invitrogen, Carlsbad, CA, USA). Copy numbers of the plasmid DNA were calculated based on their concentration and molecular weights. For each target gene, 10^8^, 10^7^, 10^6^, 10^5^, 10^4^ and 10^3^ copies/μL of plasmid DNA solutions were freshly prepared for standard samples. The qPCR reactions were run with three technical replicates using iTaq Universal SYBR Green Supermix on a Bio-Rad CFX96 touch machine (Bio-Rad, Hercules, CA, USA), and the conditions were as follows: 95°C for 5 min, followed by 40 cycles of 95°C for 30 s, 60°C for 30 s, and 72°C for 1 min, and the final melt curve for primer specificity from 65°C to 95°C with 0.5°C.

### Quantification and statistical analysis

#### Bioinformatic analysis

Following the same RNAseq pipeline detailed in Pers & Hansen (2021)^58^ and Argandona et al. (2023),^32^ raw RNA-seq reads were quality checked and trimmed with FASTQC v.0.11.8^81^ and Trimmomatic v. 0. 36^82^ with the following parameters: ILLUMINACLIP:TruSeq3-PE.fa:2:30:10 LEADING:3 TRAILING:3 SLIDINGWINDOW:4:15 MINLEN:36. The trimmed reads were aligned using HISAT v. 2.1. 0^83^ against the chromosomal assemblies of CRF-CA_Dcit for *D. citri* (GenBank: GCA_024506315.2)^21^ and isoF-IL for *B. cockerelli* (GenBank: GCA_024516035.1).^22^ The mapped reads for each gene were quantified as raw read counts using StringTie v.2.2.1^84^ using gff files annotated for each psyllid species.^21^^,^^22^ Differential expression of gene transcripts between bacteriome and body samples were determined using R (version 4.2.0)^85^ using the exact test with EdgeR.^86^ The exact test was chosen because it has been demonstrated to be more conservative compared to other tests when the number of sample replicates is smaller than five.^87^ Statistical significance of differentially expressed genes was determined with a false discovery rate (FDR) adjusted p ≤ 0.05 and ≥1.5-fold change of the normalized expression values similar to Argandona et al. (2023).^32^ In this context, “logFC” indicate log2 fold change between the groups and “logFC” ≥ 0.5849 represents significantly up-regulated genes and “logFC” ≤ −0.5849 represents significantly down-regulated genes. Gene Set Enrichment Analysis (GSEA, v4. 0. 0)^88^ was used to determine which Kyoto Encyclopedia of Genes and Genomes (KEGG) pathways are differently expressed at the normalized p ≤ 0.05 and FDR q ≤ 0.25, as described in Pers & Hansen (2021).^58^ Annotations for subsets of genes that are related to HTGs and symbiosis genes are obtained from the previous genome analyses,^22^ and further annotated here for the new genome dataset of *D. citri*-CRF using NCBI Blast and BlastKOALA.^89^ Further phylogenetic analyses for three HGTs were performed with RAxML-HPC BlackBox version 8.2.12 on the CIPRES webserver.^90^ The JTT model was employed, and RAxML was allowed to halt bootstrapping automatically. The resulting bipartition trees generated from RAxML were visualized and exported using Figtree version 1.4.4.^91^

#### *Wolbachia* endosymbiont quantification

The normalized ratio value for *Wolbachia* genome copies relative to psyllid genome copies was calculated for three biological replicates using the following equation: average *Wolbachia* DNA single gene copy quantity/average psyllid DNA single gene copy quantity based on the protocol outlined in Bookout et al. (2006).^97^ Statistical analysis of the copy number variation was conducted using an independent t-test from the ggplot2^98^ package in R version 4.1.2^85^ to determine significant (p < 0.05) differences between tissue types for the normalized ratio values for both psyllid species.

## Data Availability

• Raw RNA-seq data have been deposited at NCBI under BioProject accession number PRJNA939696. Accession numbers are also listed in the key resources table. • All code used in this paper is from the “Dataset_S9_RNAseq_Code”,^58^ which is publicly available on figshare: https://doi.org/10.25387/g3.14109851. • Any additional information required to reanalyze the data reported in this paper is available from the lead contact upon request.

## Supplemental information

Document S1. Figures S1–S3 and Table S1

Table S2. Differential expression analysis data for D. citri and B. cockerelli bacteriomes compared to body tissues using EdgeR exactTest, related to Figure 1 and 2, and Table 1 and 2

Table S3. Gene read count matrix generated via Stringtie and Ballgown for D. citri and B. cockerelli, related to Bioinformatic analysis in STAR Methods

Table S4. one-to-one orthologous gene clusters between D. citri and B. cockerelli and their expression data, related to Figure 1

Table S5. The 67 non-redundant shared orthologs out of 100 with the highest negative and positive correlations from the principal components 1 and 2 output loading matrix, related to Figure 1

Table S6. Lineage specific orthologous cluster in D. citri and B. cockerelli, related to Figure 1 and Bioinformatic analysis in STAR Methods

Table S7. Lineage-specific gene clusters in D. citri that are significantly differentially expressed in bacteriomes compared to the body tissues, related to related to Figure 1 and Bioinformatic analysis in STAR Methods

Table S8. Lineage-specific gene clusters in B. cockerelli that are significantly differentially expressed in bacteriomes compared to the body tissues, related to Figure 1 and Bioinformatic analysis in STAR Methods

Table S9. Significantly expanding gene clusters in B. cockerelli that are significantly differentially expressed in bacteriomes compared to the body tissues, related to Bioinformatic analysis in STAR Methods

Table S10. Significantly enriched pathways in bacteriomes of D. citri and B. cockerelli using GSEA, related to Figure 4

Table S11. Wolbachia genes that are potentially involved in providing nutrition, related to Figure 5
