# Supplementary material for: Unveiling metabolic integration in psyllids and their nutritional endosymbionts through comparative transcriptomics analysis
Source: iScience. 2023 Sep 15;26(10):107930. doi: 10.1016/j.isci.2023.107930 (PMC10558732; doi:10.1016/j.isci.2023.107930)
Supplement: Document S1. Figures S1–S3 and Table S1 [file mmc1.pdf]

iScience, Volume *26*

Supplemental information

Unveiling metabolic integration in psyllids and their nutritional endosymbionts through comparative transcriptomics analysis

Younghwan Kwak and Allison K. Hansen

This supplemental file includes

Supplemental Figures 1 to 3

Supplemental Table 1

Other supplemental material for this manuscript includes the following:

Supplemental Table 2-11 (provided in separate excel file)

Supplemental Figures

Supplemental Figure 1. Principal component analysis (PCA) plots of gene expression count data of *D. citri* and *B. cockerelli*, related to Bioinformatic analysis in STAR Methods.

Supplemental Figure 2. Inter-species ortholog comparison of top 67 one-to-one orthologs, related to Figure 1.

Supplemental Figure 3. Phylogenetic analysis of 16S rRNA methylation gene (*rsmJ*), related to Figure 1 and Table 2.

Supplemental Tables

Supplemental Table 1. Total RNA-Seq reads sequenced, quality trimmed, and successfully mapped as pairs for each bacteriome and body tissue, related to Bioinformatic analysis in STAR Methods.

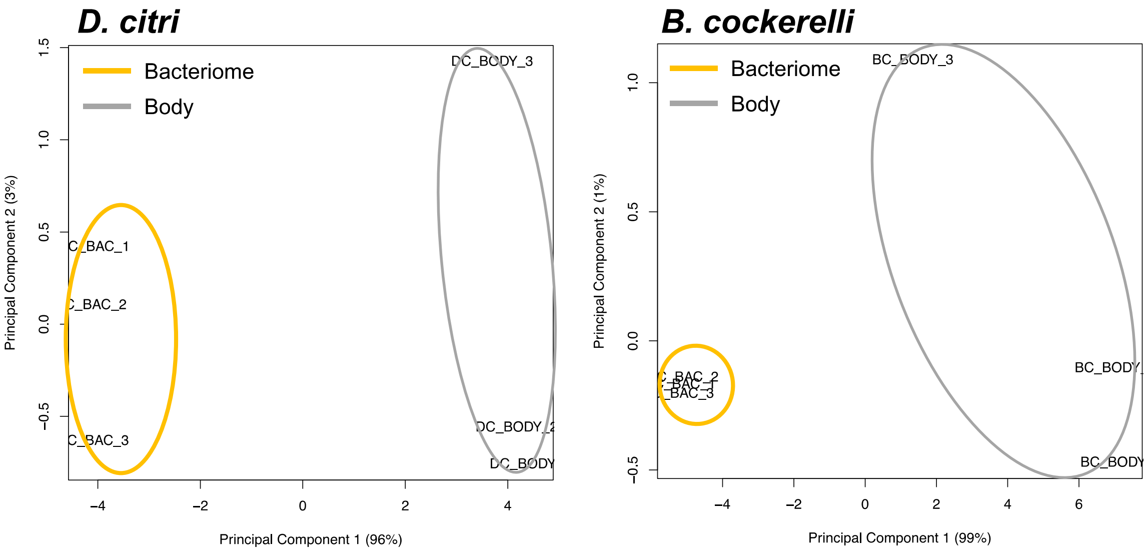

Supplemental Figure 1. Principal component analysis (PCA) plots of gene expression count data of *D. citri* (left) and *B. cockerelli* (right), related to Bioinformatic analysis in STAR Methods. Two tissue types are color-coded as indicated in inset legends. The variance of principle components 1 and 2 are shown in axis labels where most of the variation among samples are explained on the X-axis.

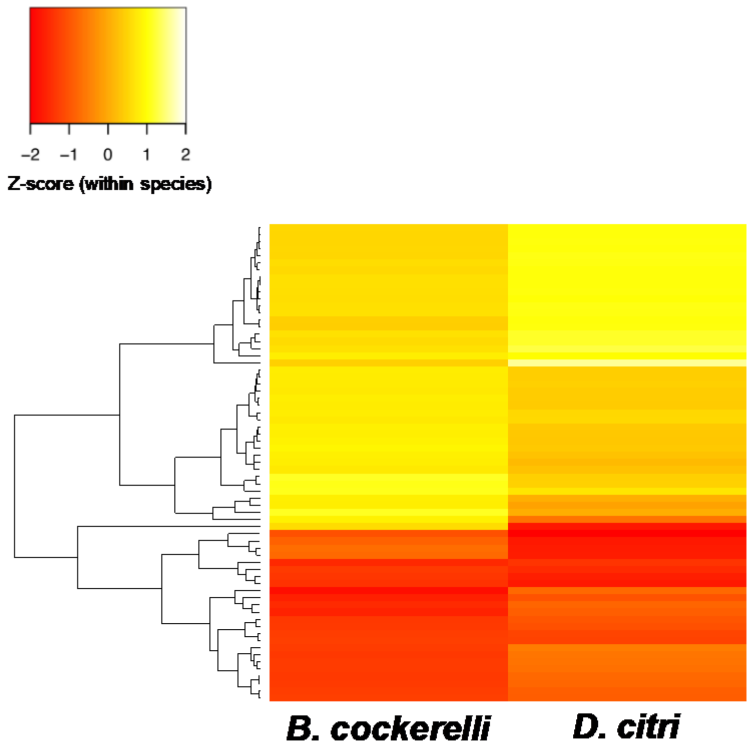

Supplemental Figure 2. Inter-species ortholog comparison of top 67 one-to-one orthologs with the greatest variance in standardized Log fold-change expression of bacteriomes compared to body tissues in *B. cockerelli* and *D. citri,* related to Figure 1. Each row represents a shared one-to-one ortholog between *B. cockerelli* and *D. citri*. Legend color indicates Z-score range, which was calculated within species based on Log Fold change of bacteriome relative to body; positive Z-scores indicate genes up-regulated in bacteriomes compared to body and negative scores indicate genes down-regulated in bacteriomes compared to body. See Supplemental Table 10 for detail on each one-to-one ortholog, its annotations, and fold change for each species.

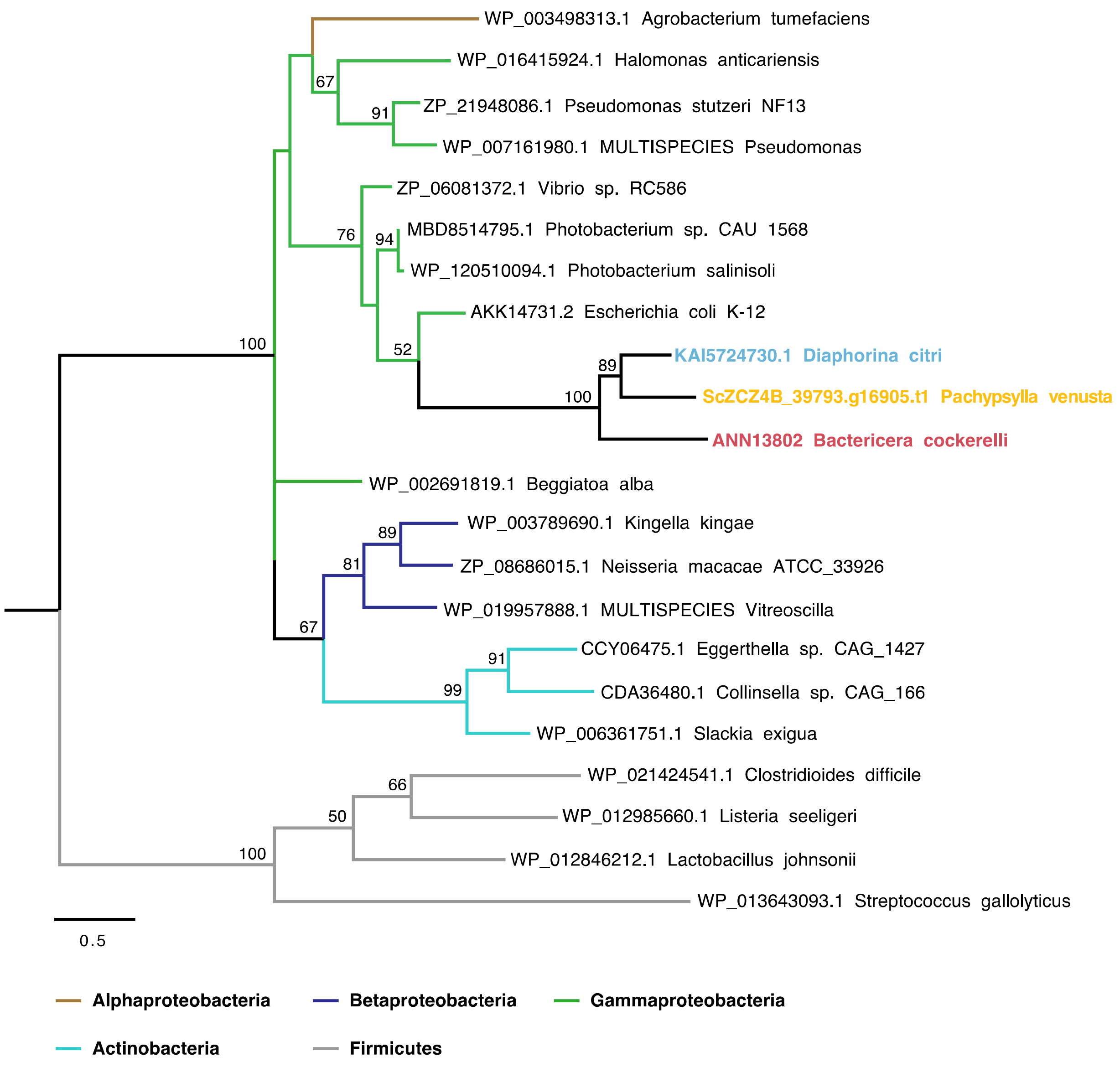

Supplemental Figure 3. Phylogenetic analysis of 16S rRNA methylation gene (*rsmJ*), related to Figure 1 and Table 2. The branch labels in bold indicate psyllid genes with their gene IDs. Branches are colored according to their bacterial classification. Only branch support at 50% or above is shown. The tree was rooted with the outgroup Firmicutes.

RNA-Seq samples	Total reads	Total reads after trimming	Paired reads	Unpaired reads	Overall alignment rate
D. citri					
DC1 bacteriome	22,896,867	22,845,698	17,972,453	4,873,245	75.08%
DC1 Body Cells	25,122,905	25,064,597	19,591,642	5,472,955	90.46%
DC2 bacteriome	24,247,574	24,198,848	18,946,200	5,252,648	75.67%
DC2 Body Cells	23,142,913	23,096,306	18,207,315	4,888,991	87.60%
DC3 bacteriome	26,870,276	26,820,659	20,820,494	6,000,165	74.62%
DC3 Body Cells	25,217,218	25,167,628	19,609,977	5,557,651	90.28%
B. cockerelli					
BC1 bacteriome	23,382,333	23,330,084	18,164,402	5,165,682	81.08%
BC1 Body Cells	26,044,014	25,994,878	20,443,187	5,551,691	76.71%
BC2 bacteriome	27,205,923	27,154,811	21,112,520	6,042,291	80.32%
BC2 Body Cells	26,886,380	26,832,108	21,351,764	5,480,344	76.96%
BC3 bacteriome	28,671,247	28,612,318	22,615,603	5,996,715	80.56%
BC3 Body Cells	31,033,239	30,971,386	24,435,597	6,535,789	76.37%

Supplemental Table 1. Total RNA-Seq reads sequenced, quality trimmed, and successfully mapped as pairs for each bacteriome and body tissue, related to Bioinformatic analysis in STAR Methods.
